# IDENTIFYING ASSISTED LIVING SAFETY PRIORITIES: A DELPHI PANEL WITH RESIDENT, FAMILY, AND PROFESSIONAL STAKEHOLDERS

**DOI:** 10.1093/geroni/igac059.1615

**Published:** 2022-12-20

**Authors:** Cassandra Dictus, Youngmin Cho, Victoria Bartoldus, Matthias Hoben, Stephanie Chamberlain, Anna Beeber

**Affiliations:** University of North Carolina - Chapel Hill, Chapel Hill, North Carolina, United States; University of North Carolina - Chapel Hill, Chapel Hill, North Carolina, United States; University of North Carolina - Chapel Hill, Chapel Hill, North Carolina, United States; University of Alberta, Edmonton, Alberta, Canada; University of Alberta, Edmonton, Alberta, Canada; Johns Hopkins University, Baltimore, Maryland, United States

## Abstract

While assisted living (AL) communities emphasize resident safety, one barrier to resident safety is a lack of information about AL stakeholders’ safety priorities. As part of a larger research project to create a toolkit to foster resident and family engagement in safety in AL, we created a stakeholder panel that includes 13 AL residents, family members, and professionals (i.e., direct care workers, administrators, researchers, and policymakers). This paper describes a web-based Delphi process to create a ranked safety priority list with stakeholders. After three rounds involving online surveys and Zoom group discussions, the stakeholder group came to a consensus on a final list of 14 ranked safety priorities. Using verbatim transcripts of the Zoom discussions and chat, we conducted content analysis to highlight the rationales for the 14 ranked safety priorities on the final list. Reasons to prioritize safety concerns included the seriousness of the impact on AL residents and system-level root causes. These findings will be used to guide the development of a toolkit to improve resident and family engagement in the safety of AL. This list can also help AL communities and researchers at large to better understand what safety priorities are most important to a broad range of AL stakeholders and why.

